# Diagnostic Accuracy and Generalizability of a Deep Learning-Based Fully Automated Algorithm for Coronary Artery Stenosis Detection on CCTA: A Multi-Centre Registry Study

**DOI:** 10.3389/fcvm.2021.707508

**Published:** 2021-11-05

**Authors:** Lixue Xu, Yi He, Nan Luo, Ning Guo, Min Hong, Xibin Jia, Zhenchang Wang, Zhenghan Yang

**Affiliations:** ^1^Affiliated Beijing Friendship Hospital, Capital Medical University, Beijing, China; ^2^Shukun (Beijing) Technology Co., Ltd., Beijing, China; ^3^Department of Computer Software Engineering, Soonchunhyang University, Asan-si, South Korea; ^4^Faculty of Information Technology, Beijing University of Technology, Beijing, China

**Keywords:** coronary artery disease, computed tomographic angiography, deep learning, invasive coronary angiography (ICA), diagnostic test

## Abstract

**Aims:** In this retrospective, multi-center study, we aimed to estimate the diagnostic accuracy and generalizability of an established deep learning (DL)-based fully automated algorithm in detecting coronary stenosis on coronary computed tomography angiography (CCTA).

**Methods and results:** A total of 527 patients (33.0% female, mean age: 62.2 ± 10.2 years) with suspected coronary artery disease (CAD) who underwent CCTA and invasive coronary angiography (ICA) were enrolled from 27 hospitals from January 2016 to August 2019. Using ICA as a standard reference, the diagnostic accuracy of the DL algorithm in the detection of ≥50% stenosis was compared to that of expert readers. In the vessel-based evaluation, the DL algorithm had a higher sensitivity (65.7%) and negative predictive value (NPV) (78.8%) and a significantly higher area under the curve (AUC) (0.83, *p* < 0.001). In the patient-based evaluation, the DL algorithm achieved a higher sensitivity (90.0%), NPV (52.2%) and AUC (0.81). Generalizability analysis of the DL algorithm was conducted by comparing its diagnostic performance in subgroups stratified by sex, age, geographic area and CT scanner type. The AUCs of the DL algorithm in the aforementioned subgroups ranged from 0.79 to 0.86 and from 0.75 to 0.93 in the vessel-based and patient-based evaluations, both without significant group differences (*p* > 0.05). The DL algorithm significantly reduced post-processing time (160 [IQR:139–192] seconds), in comparison to manual work (*p* < 0.001).

**Conclusions:** The DL algorithm performed no inferior to expert readers in CAD diagnosis on CCTA and had good generalizability and time efficiency.

## Introduction

Coronary computed tomography angiography (CCTA) is a non-invasive tool with a high diagnostic accuracy and negative predictive value (NPV) in the estimation of coronary narrowing (1). Nevertheless, the CCTA examination workflow is time consuming and labor intensive, with an average post-processing and reporting time ≥30 min (2). With a growing number of coronary artery disease (CAD) patients (3, 4), the supply-demand imbalance of CCTA has become a growing problem. Therefore, the acceleration of the current CCTA workflow is imperative.

Deep learning (DL) has been used to assist in the imaging interpretation of CAD (5, 6), incorporating the risk stratification of patients (7–9) and the segmentation and quantification of cardiac and coronary structures (10–12). The generalizability of DL-based models is of increased importance because overfitted models could hardly be applied in real-world clinical practice. Recently, we developed a DL-based fully automated algorithm to streamline CCTA reconstruction and interpretation workflows and found that the DL algorithm significantly improved the time efficiency and diagnostic consistency of CCTA (13, 14). In addition, by using invasive coronary angiography (ICA) as a standard reference, the accuracy of the DL algorithm was not inferior to that of expert readers. However, the CCTA data were acquired from a single center with only one or two types of computed tomography (CT) scanners, and the diagnostic performance and reproducibility of the DL algorithm still need to be evaluated.

Accordingly, we used a completely external multi-center dataset to estimate the diagnostic accuracy and generalizability of the DL algorithm in comparison to ICA. The CCTA data were obtained from 27 sites (across 5 geographic areas), 4 types of vendors and 5 brands of CT scanners. There are two aims of our study: (a) to compare the diagnostic accuracy of the DL algorithm with that of expert readers in a larger sample; and (b) to determine whether the DL algorithm performs robustly for data obtained from patients with different ages, sexes, and geographic information and for data acquired from different types of CT scanners.

## Methods

### Study Design and Datasets

Patients with suspected CAD from 27 hospitals and 5 geographic areas (Northeast, Northwest, South, North and East China) (Supplementary Table 1) were retrospectively enrolled between January 2017 and August 2019. The study was registered at the Chinese Clinical Trial Registry (ChiCTR1900021867), and the protocol was approved by the local institutional review boards of each of the 27 enrolling hospitals in China, and the informed consent was waived. Each participating hospital incorporated CCTA and ICA into daily clinical practice. The inclusion criteria were the accomplishment of CCTA followed by ICA within 6 months. The exclusion criteria were as follows: missing CCTA or ICA data, history of coronary artery bypass grafting or stenting, coronary anomalies, poor image quality of ICA data or young age (< 18 years).

In accordance with the Society of Cardiovascular Computed Tomography (SCCT) guidelines or each site's institutional policy (15), all image acquisition and image post-processing for CCTA and ICA data were performed with no restrictions on the CT scanner type or the type of iodinated X-ray contrast. All the CCTA data were acquired on CT scanners of 64-detector rows or greater of 5 scanner brands: GE Medical Systems (Discovery CT750, Revolution CT), Philips Medical Systems (iCT), Siemens Healthineers (SOMATOM Force, SOMATOM Definition Flash, SOMATOM Definition AS+, Biograph), Toshiba (Aquilion ONE) and Shanghai United Imaging Healthcare (UIH uCT760) (Supplementary Figure 1). The CT scanner type differed by center. The type of electrocardiographic gating method was defined as either retrospective helical gating or prospective axial triggering. The tube potential (kV) ranged from 70 to 140 kV (Table 1 and Supplementary Table 2). Datasets were reconstructed retrospectively with iterative reconstruction and electrocardiography editing when necessary. The phase with optimal image quality was used for further CCTA analysis.

**Table 1:** Baseline information.

**Variable**	**All patients (*n* = 527)**
**Sex**	
Male	353/527 (67.0%)
Female	174/527 (33.0%)
**Age** (years)	62.2 ± 10.2
**HR** (bpm)	65.9 ± 14.3
**BMI** (kg/m^2^)	24.6 ± 3.0
**Hypertension**	310/507 (61.1%)
**Hyperlipidemia**	79/456 (17.3%)
**DM**	133/497 (26.8%)
**Smoking**	207/486 (42.6%)
**CAD history**	24/513 (4.7%)
**Tube potential**	
≤80 kV	17/527 (3.2%)
90 ~ 110 kV	178/527 (33.8%)
≥120 kV	332/527 (63.0%)
DLP (mGy × cm)	544.0 [298.1–974.5]
**Numbers of diseased vessels on ICA**	
LM disease	12
0 vessel disease	92
1 vessel disease	210
2 vessel disease	170
3 vessel disease	55

*Data are n/N (% of non-missing data), mean (SD) or median [interquartile range].*

*HR, hear rate; BMI, body mass index; DM, diabetes mellitus; CAD, coronary artery disease; DLP, dose length product; ICA, invasive coronary angiography; LM, left main*.

### Manual Post-processing and Visual Assessment of Coronary Stenosis

**Figure 1:**
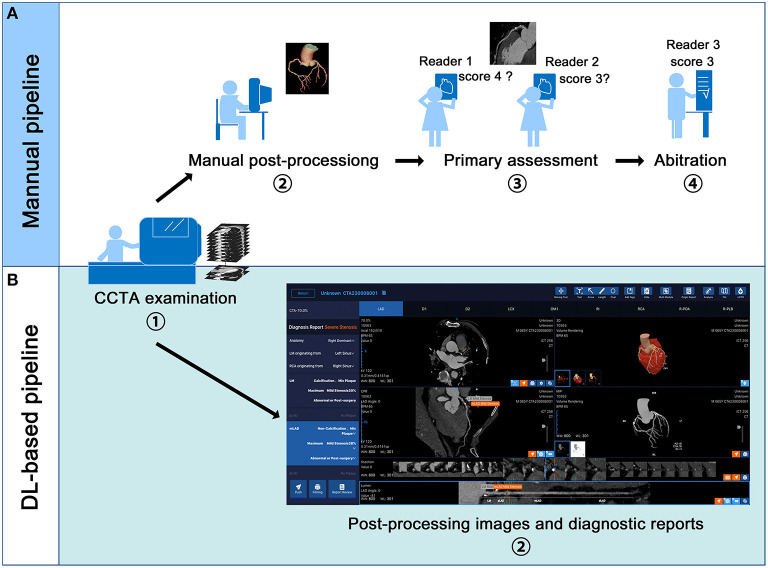
Manual and deep learning (DL)-based fully automated pipeline of coronary computed tomography angiography (CCTA) examination and interpretation. **(A,B)** displays the manual and DL-based pipeline respectively. In the DL-based pipeline, the volume rendering, curve plannar reformation, maximum intensity projection and axial images are automatically presented. The stenosis reports including the position of the lesion, the plague types and percentage of stenosis are also automatically displayed.

Reformats images including maximum intensity projection (MIP), multi-planar reformation (MPR), curved planar reformation (CPR) and volume rendering (VR) were obtained on a on an image analysis workstation (GE Advantage Workstation 4.7, GE Healthcare, Milwaukee, Wisconsin). The interpretation of the CCTA was performed by 10 board-certified radiologists, with experience in judging more than 5,000 CCTA scans. The 10 readers were blinded to the clinical history of the enrolled patients, and they were not involved in the patients' clinical assessment. The 10 readers were divided evenly into 5 groups, and all the anonymous scans were distributed randomly among the 5 groups. For any group, each of the 2 readers independently evaluated the anonymized and randomly ordered scans and was blinded to the ICA results of the enrolled patients. The image quality of each segment was estimated by a Likert 5-scale score: a score of 5 indicated excellent quality (absence of artifacts associated with motion or coronary calcification); a score of 4 indicated good quality (very mild artifacts); a score of 3 indicated moderate quality (minor artifacts); score of 2 was still considered diagnostic quality (considerable artifacts but maintained visualization of arterial lumen); and a score of 1 indicated non-diagnostic quality (with severe motion artifacts or extensive wall calcification). The coronary artery tree was visually evaluated based on an SCCT 18-segment model (16) by using axial sections and curved multi-planar reformations along the vessel centreline for all segments with image quality scores of ≥1 and without severe calcification. According to the Coronary Artery Disease Reporting and Data System (CAD-RADS) (17) guidelines, luminal diameter stenosis severity was assessed in segments with a diameter of 1.5 mm or greater. The manual pipeline of post-processing and stenosis assessment is summarized in Figure 1A.

All CCTA findings were compared with the corresponding ICA results. The anonymized and randomly ordered ICA scans were evaluated by another 10 cardiologists with experience in judging more than 3,000 ICA scans. Following the same stenosis grading scale as CCTA, 10 independent and blinded readers were distributed evenly into 5 groups, and each group graded the coronary stenosis from ICA scans for segments with a diameter of 1.5 mm or greater. In the primary analysis, at least 50% diameter stenosis was defined as obstructive CAD for both CCTA and ICA. The secondary analysis defined a cutoff of ≥70% diameter stenosis for CCTA and ICA. And this analysis was only used to investigate the generalizability of the DL-algorithm.

If 2 readers in the same group failed to achieve a unanimous agreement for a CCTA or ICA finding, the consensus was made by either a CCTA arbitration panel consisting of 2 radiologists with experience in judging more than 8,000 CCTA scans or an ICA arbitration panel consisting of 2 cardiologists with experience in judging more than 8,000 ICA scans.

### Automated Post-processing and Assessment

**Figure 2:**
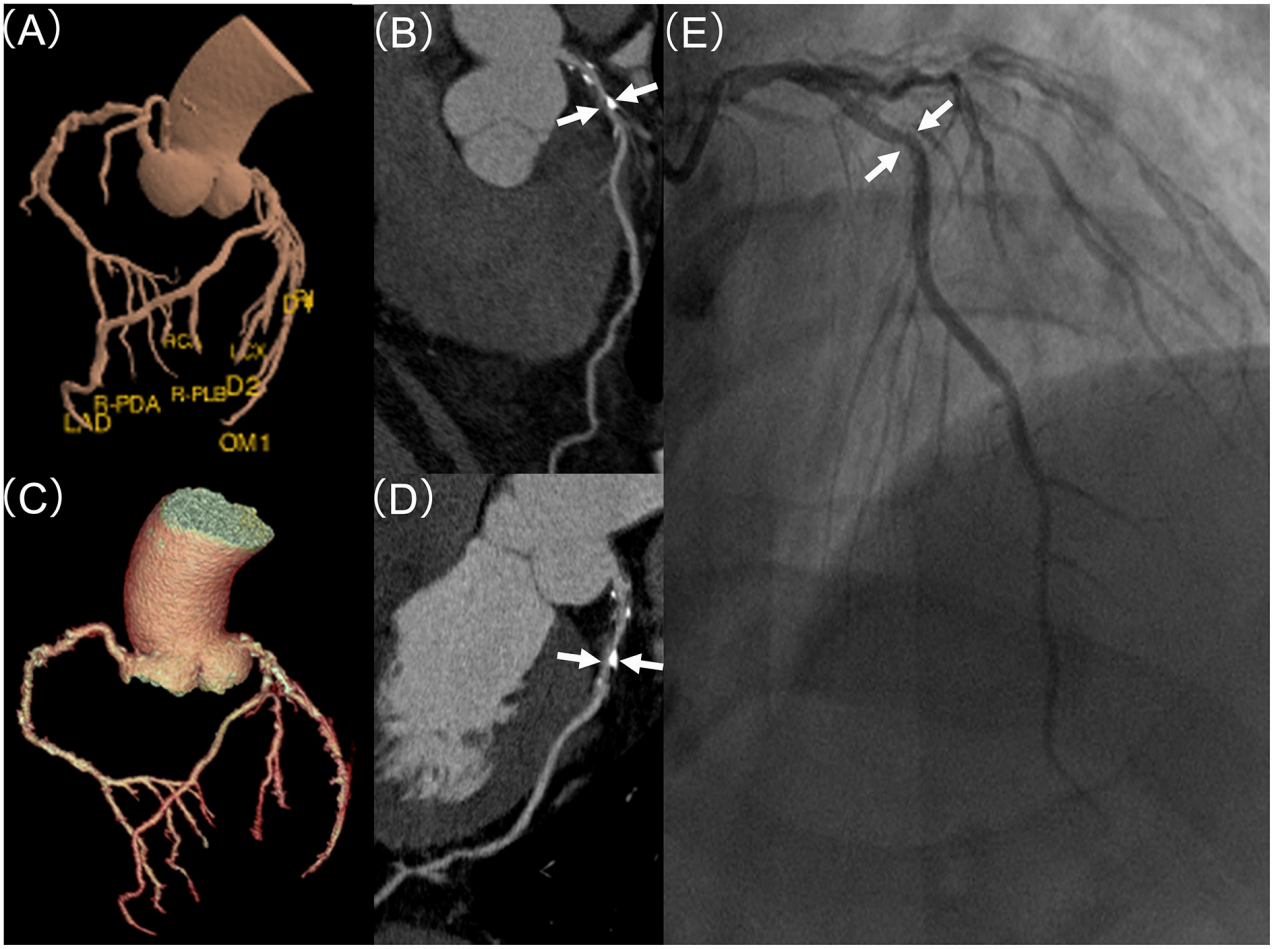
Coronary stenosis detection of expert readers vs. the deep learning (DL)-based fully automated algorithm, in comparison to invasive coronary artery (ICA). **(A)** demonstrates DL-based and **(C)** displays manual based volume rendering image of a coronary tree, respectively. **(B,D)** displays curve planner reformation image of left anterior descending (LAD) with stenosis (white arrow) based on DL or manual post-processing, respectively. **(E)** shows the lesion (white arrow) of LAD in invasive coronary angiography with a mild (25–49%) stenosis.

We used a previous-reported DL algorithm (13) to achieve automatic vascular extraction and stenosis assessment (Figures 1B, 2). The DL algorithm (CoronaryDoc, ShuKun Techonolgy, Beijing) can be divided into three parts: coronary vascular segmentation, coronary artery branches and segments identification and stenosis detection (Supplementary Figure 2). A total of 9425 retrospectively collected CCTA data from 45 hospitals in China were used for training (70%), tuning (20%) and validating (10%) the DL algorithm. Image quality evaluation and image annotation were performed in a data center, where 32 board-certified radiologists joined the work.

An improved 3-dimensional (3D) U-Net was used for coronary tree segmentation (18) which combined with a Bottle-Neck design for coronary arteries and aorta segmentation, and a connected growth prediction model (CGPM) for solving the problem of vascular segmentation fracture. The 3D U-net architecture was trained using the stochastic gradient descent (SGD) optimizer with a momentum of 0.95, a peak learning rate of 0.1 for randomly initialized weights, a weight decay of 0.0001, and an initial learning rate of 0.01 that shrank by 0.99995 after each training step of 200,000 iterations. Dice loss was used to evaluate the model performance (19). The model with the lowest Dice loss on the validation set was selected.

With the segmented coronary tree, the corresponding centrelines were generated using a 3D skeleton extraction algorithm (20). Reformat images including MIP, MPR, CPR and VR were automatically obtained. A fully automatic identification algorithm for coronary arteries based on SCCT 18-segment model was applied to identify the branches and segments of each coronary artery (16). In stenosis detection, V-net Fully Convolutional Neural Networks for Volumetric Medical Image Segmentation (V-net) was used (13). Atherosclerotic plaque can be classified into calcified plaque, non-calcified plaque, and mixed plaque according to its composition. Due to the different CT density of the different plagues, two 2D V-net models were trained to detect the calcified plaque on CPR and the non-calcified and mixed plaques on straightened MPR, respectively. Stenosis along the long axis of the vessel was calculated based on the radius of the plaque and the radius of upstream and downstream blood vessels.

### Statistical Analysis

Statistical analysis was performed using SPSS (version 26.0, SPSS Inc., Chicago, USA) and MedCalc (version 19.0.7, MedCalc Software bvba, Ostend, Belgium). Continuous normally distributed variables are described using the mean ± SD, while not normally distributed variables are presented as median (quartiles). Categorical descriptive data are described as numbers (percentages).

To compensate for the bias caused by (a) simply regarding non-diagnostic results as either positive or negative results or (b) the exclusion of non-diagnostic data, multiple imputation was applied to impute the missing data of the non-diagnostic results in the visual assessment. The variables of sex, age, area, rows of detectors, and brand type with complete information and available visual assessment outcomes of CCTA were used in multiple imputation models. In addition, a sensitivity analysis was conducted by using the 3 × 2 table method to classify non-diagnostic results either as “false negative” or “false positive” (21) (Supplementary Figure 3).

Receiver operating characteristic analysis was used to compare the diagnostic performance of the DL algorithm and human experts, using ICA as a standard reference. For a ≥50% stenosis segment with a diameter of ≥1.5 mm, the sensitivity, specificity, positive predictive value (PPV), NPV, and AUC with 95% confidence interval (CI) were calculated by the standard methods (22). AUCs were compared using the non-parametric approach of DeLong and colleagues (23). Only vessel-based and patient-based results were evaluated because they were the most clinically meaningful.

To estimate the generalizability of the DL algorithm, AUCs were compared in sex-, age-, geographic- and scanner type-based subgroups.

Time comparison of the DL algorithm vs. manual work was performed by using Wilcoxon signed-rank test.

## Results

### Study Participants

**Figure 3:**
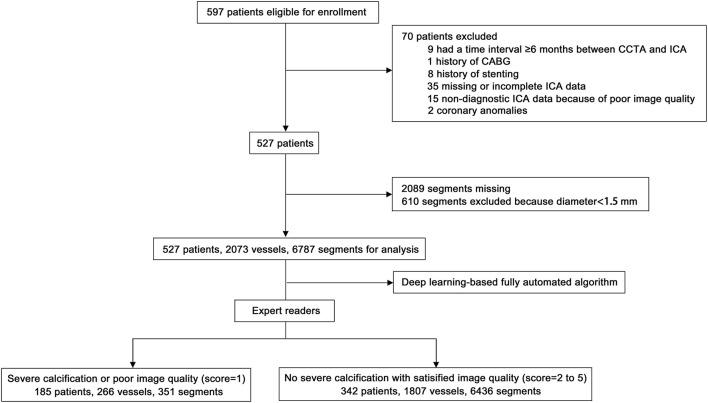
Flow chart of patient enrolment. CCTA, coronary computed tomography angiography; ICA, invasive coronary angiography; CABG, coronary artery bypass grafting.

Five hundred ninety-eight patients were enrolled. A total of 70 patients were excluded for the following reasons: 9 patients had a time interval ≥6 months between CCTA and ICA, 1 patient had a history of coronary artery bypass grafting (CABG), 8 patients had a history of stenting, 50 patients had incomplete or non-diagnostic ICA data, and 2 patients had coronary anomalies. In addition, 2,089 segments were missing, and 610 segments were excluded because they had diameter <1.5 mm. Therefore, 527 patients with 2,073 vessels and 6,787 segments were included in the analysis (Figure 3).

Table 1 summarizes the demographic characteristics of the analysis cohort of 527 patients. Overall, in this cohort, the mean age was 62.2 ± 10.2 years, and 33.0% were females. The vessel-based prevalence of obstructive CAD was 40.3%, and the patient-based prevalence was 83.5%.

### Vessel-Based Comparison of the Diagnostic Accuracy of the DL Algorithm vs. Expert Readers

The sensitivity of the DL algorithm was 65.7 (CI 62.4–68.9%), which was higher than that of the expert readers, which was 58.6 (CI 57.2–60.1%). The NPV of the DL algorithm (78.8%; CI 76.6–81.0%) was also higher than that of the experts (76.7%; CI 75.7–77.6%). However, the specificity and PPV of the DL algorithm were lower. Using the 3 × 2 table method, the additional sensitivity analysis showed that the DL algorithm performed better than the expert readers in terms of sensitivity [DL algorithm 65.7% (CI 62.4–68.9%) vs. experts 51.3% (CI 47.9–54.5%)] and NPV [DL algorithm 78.8% (CI 76.6–81.0%) vs. experts 73.0% (CI 70.7–75.3%)], while the specificity of the DL algorithm was lower. The PPVs of the DL algorithm and experts were similar (Table 2).

**Table 2:** Diagnostic accuracy of expert readers vs. deep-learning based fully automated (DL) algorithm.

	**Expert readers**		**DL algorithm**
	**(with imputation method)^a^**	**(with the 3 × 2 table method)^b^**	
**Vessel-based evaluation**			
Sensitivity (95% CI)	58.6 (57.2–60.1%)	51.3 (47.9–54.5%)	65.7 (62.4–68.9%)
Specificity (95% CI)	92.2 (91.5–92.9%)	88.9 (87.1–90.5%)	85.6 (83.6–87.6%)
PPV (95% CI)	83.6 (82.3–84.9%)	75.6 (72.3–79.0%)	75.5 (72.4–78.5%)
NPV (95% CI)	76.7 (75.7–77.6%)	73.0 (70.7–75.3%)	78.8 (76.6–81.0%)
**Patient-based evaluation**			
Sensitivity (95% CI)	84.0 (82.5–85.7%)	53.6 (48.4–58.6%)	90.0 (86.8–92.7%)
Specificity (95% CI)	71.0 (66.7–75.4%)	59.8 (49.4–70.1%)	55.2 (43.7–66.7%)
PPV (95% CI)	93.6 (92.6–94.7%)	87.1 (83.0–90.4%)	91.0 (88.3–93.6%)
NPV (95% CI)	46.7 (43.0–50.5%)	20.3 (15.2–25.4%)	52.2 (42.4–62.0%)

a *indicates the results of the imputed visual assessment.*

b *means the results of the visual assessment with the 3 × 2 table method, which classifies non-diagnostic results as “false negative” or “false positive.”*

*PPV, positive predictive value; NPV, negative predictive value; CI, confidence interval*.

**Figure 4:**
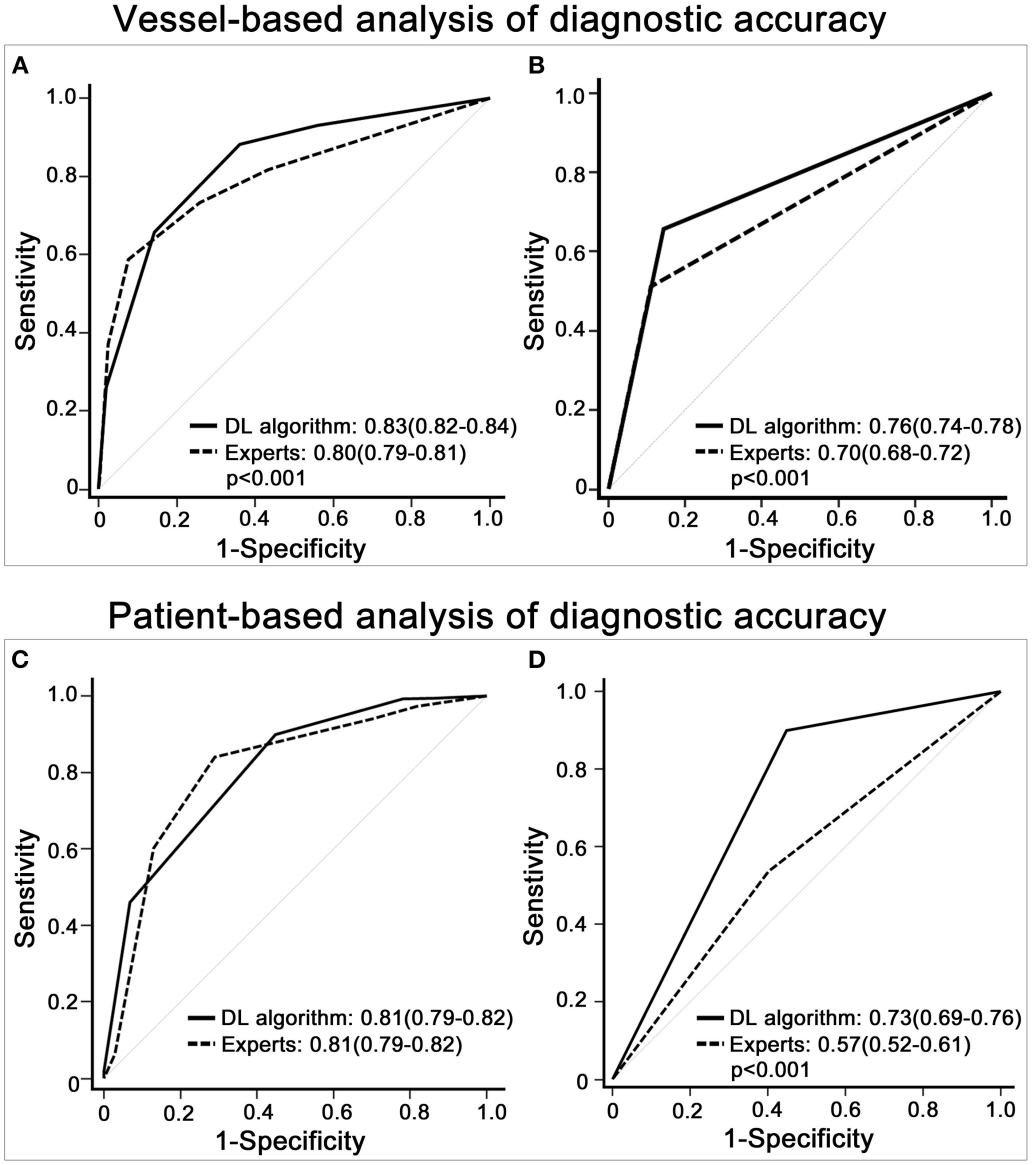
Diagnostic accuracy of expert readers vs. the deep learning (DL)-based fully automated algorithm. **(A)** shows that the receiver operating characteristic (ROC) curve of the DL algorithm was higher than that of the imputed visual assessment results in the vessel-based evaluation. **(B)** displays the ROC curve of the DL algorithm and that of the expert readers with the 3 × 2 table method in the vessel-based evaluation. **(C,D)** depicts patient-based group comparisons between the DL algorithm and expert readers, **(C)** shows the results of the multiple imputation data, and **(D)** demonstrates the outcomes of the 3 × 2 table method. The results with significant group differences are shown with *p*-values.

The AUC of the DL algorithm was 0.83 (CI 0.82–0.84), which was significantly higher than that of the expert readers, with an AUC of 0.80 (CI 0.79–0.81) (*p* < 0.001). In the additional sensitivity analysis, the DL algorithm achieved an AUC of 0.76 (CI 0.74–0.78) compared with 0.70 (CI 0.68–0.72) for the expert readers (*p* < 0.001) (Figures 4A,B).

### Patient-Based Comparison of the Diagnostic Accuracy of the DL Algorithm vs. Expert Readers

In the comparisons of the imputed visual evaluations, the sensitivity of the DL algorithm was higher [DL algorithm 90.0 (CI 86.8–92.7%) vs. experts 84.0 (CI 82.5–85.7%)], while the specificity and PPV of the DL algorithm were lower. The NPV of the DL algorithm was 52.2 (CI 42.4–62.0%), slightly higher than that of the experts (46.7; CI 43.0–50.5%). In the additional sensitivity analysis, the DL algorithm had higher sensitivity [DL algorithm 90.0 (CI 86.8–92.7%) vs. experts 53.6 (CI 48.4–58.6%)] and NPV [DL algorithm 52.20 (CI 42.4–62.0%) vs. experts 20.3 (CI 15.2–25.4%)] than the expert readers. Regarding specificity, the DL algorithm and experts were similar, and regarding PPV, the DL algorithm performed better (Table 2).

The AUC of the DL algorithm was 0.81 (CI 0.79–0.83), which was the same (0.81; CI 0.78–0.83) for the imputed visual evaluation. By using the 3 × 2 table method, the AUC of the DL algorithm was significantly higher [DL algorithm 0.73 (CI 0.67–0.78) vs. experts 0.57 (CI 0.51–0.62)] (*p* < 0.001) (Figures 4C,D).

### Generalizability of the DL Algorithm

**Figure 5:**
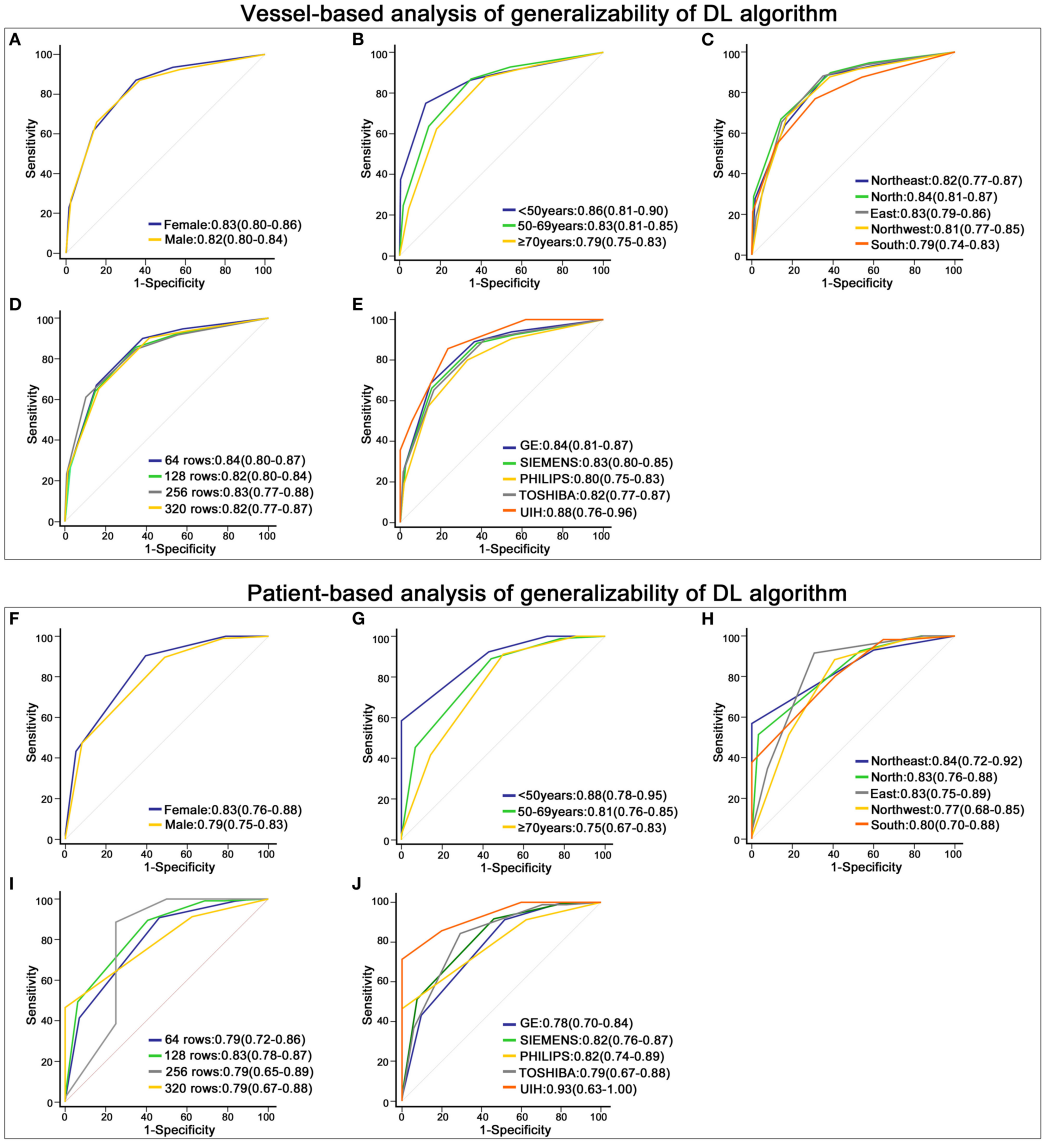
Robustness of the deep learning (DL)-based fully automated algorithm. **(A–E)** displays the receiver operating characteristic (ROC) curve of the DL algorithm in different subgroups at the vessel-based level. **(A)** shows the results of males and females, **(B)** is the outcome of patients with different ages, **(C)** depicts the results of patients from different geographic areas, and **(D,E)** display the outcomes of data acquired on different rows of detectors and different brands of CT scanners. **(F–J)** shows the ROC curve of the DL algorithm in groups stratified by sex **(F)**, age **(G)**, geographic areas **(H)**, rows of detectors **(I)** and brands of CT scanners **(J)** at the patient-based level.

For vessel-based evaluation, the DL algorithm had similar AUCs in (a) the sex-based subgroups (male and female), (b) the age-based subgroups (<50 years, 50–69 years and ≥70 years), (c) the geographic-based subgroups (Northeast, Northwest, South, North and East China), (d) the subgroups of different detector rows (64 rows, 128 rows, 256 rows and 320 rows) and (e) the subgroups of different CT scanner brands (GE, Siemens, Philips, Toshiba and UIH) (*p* > 0.05) (Figures 5A–E).

For the patient-based evaluation, the diagnostic performance of the DL algorithm remained robust in different subgroups (*p* > 0.05) (Figures 5F–J).

When using ≥70% stenosis a cutoff, the DL algorithm had similar AUCs in different subgroups (*p* > 0.05) (Supplementary Figures 3, 4).

### Time Comparison of the DL Algorithm vs. Manual Post-processing

The median post-processing time by manual work was 837 [IQR:609–1,065] seconds, while DL algorithm (160 [IQR:139–192] sec) significantly reduced the post-processing time (*p* < 0.001).

## Discussion

In this multi-center study, we used a completely external dataset to validate the diagnostic accuracy and generalizability of the DL algorithm. We found that (a) the DL algorithm performed no inferior to experts with higher sensitivity, NPV and AUC; (b) the DL algorithm performed robustly in different subgroups stratified by sex, age, geographic area, rows of detectors and brands of CT scanners; (c) the DL algorithm significantly reduced time cost.

Using ICA as a standard reference, two studies compared the diagnostic accuracy of the DL algorithm and expert readers. One reported that the DL algorithm outperformed expert readers with a vessel-based AUC of 0.87 (13), and the other showed that the DL algorithm performed equally to expert readers (14). Several limitations underlie these findings: (a) single-center designs with a small sample size, (b) CCTA data were acquired from only 1 or 2 types of CT scanners and (c) biases caused by excluding segments with poor image quality (21, 24). Several other DL-based automated algorithms have proven useful in distinguishing stenotic coronary arteries (25, 26). However, these results were obtained by using human readers' outcomes rather than ICA as standard references. Our evaluation of the diagnostic accuracy of the DL algorithm was based on a multi-center (27 sites in 5 geographic areas) and multi-vendor (4 types of detector rows and 5 brands of CT scanners) dataset. Instead of excluding segments with poor image quality or classifying non-evaluable results as either positive or negative, we used multiple imputation and the 3 × 2 table method to deal with non-diagnostic segments for visual assessment, avoiding the biased overestimation of diagnostic accuracy (21). Because the 3 × 2 table method classified non-diagnostic results as “false” results, the diagnostic accuracy based on the 3 × 2 table method was poorer than that based on multiple imputation. Compared with expert readers, the diagnostic performance of the DL algorithm was better with reliable accuracy for the diagnosis of obstructive CAD. The AUCs of the DL algorithm were 0.83 and 0.81 at the vessel and patient levels, respectively.

As has been well elucidated, the vital clinical value of CCTA, which has a non-invasive nature and high NPV, is to rule out patients without obstructive CAD. Most previous CCTA studies were performed on populations with a low (~20%) to intermediate (~50%) prevalence of disease (1, 27, 28). NPV is influenced by the prevalence of disease. Under an 82.4% prevalence of CAD, a meta-analysis found that the NPV of CCTA dropped to 42.1% (28). In our study, the 83.5% patient prevalence of CAD decreased the NPVs of both the expert readers and the DL algorithm. The NPV of the DL algorithm was 52.2%, which was still higher than that of the expert readers. Our findings indicated that the DL algorithm had a better rule-out ability than visual inspection in patients with a high prevalence of CAD.

Generalizability is a great challenge for DL-based models. If a model performs well in only a selected population, it could hardly be applied in clinical practice. To test the generalizability of the DL algorithm, we compared its diagnostic accuracy in different subgroups. When ≥50% luminal stenosis was diagnosed as CAD, the AUCs of the DL algorithm varied from 0.79 to 0.88 at the vessel level and from 0.75 to 0.93 at the patient level. Using ≥70% luminal stenosis as a cutoff, the AUCs of the DL algorithm varied from 0.82 to 0.90 at the vessel level and from 0.77 to 0.91 at the patient level. However, no significant differences were found among patients with different ages, sexes or geographic areas or among data acquired from different CT scanners. The outcome validated the robust performance of the DL algorithm and implied that the DL algorithm could work for patients in most hospitals.

Post-processing and interpreting CCTA results are time consuming and labor intensive. Fatigue from grading large numbers of images and the subjectivity of image interpretations usually result in non-negligible intra- and inter-reader variability. The large and increasing CAD populations as well as insufficient high-quality medical resources are deteriorating the supply-demand imbalance (29). Previous studies compared the post-processing and diagnostic time between the DL algorithm and humans and found that the DL algorithm displayed outstanding time efficiency (saving >80% time) (13, 14). In our study, the DL algorithm performed faster and no inferior to expert readers, and the DL algorithm remained robust in diagnosing CAD. Therefore, the DL algorithm will potentially improve the current CCTA workflow by reducing the time cost, promoting diagnostic consistency and retaining high diagnostic accuracy. The DL algorithm will benefit both patients and clinicians in several aspects. First, in the clinical scenario of a large hospital, patients who undergo CCTA will receive good services faster. For small health centers that lack professional CCTA clinicians or radiologists, the DL algorithm can supply patients with a reliable primary CCTA diagnosis. For clinicians, their accuracy and efficiency will be improved by the assistance of the DL algorithm because of the faster and more consistent detection of CAD. However, it is still worthwhile to consider how to best combine the strengths of the DL algorithm and clinicians to optimize the accuracy and efficiency of CCTA.

Our study has several limitations. Firstly, the prevalence of CAD in our study was very high, thus decreasing NPVs. A prospective study is needed to test the rule-out ability of the DL algorithm in a larger population with a lower prevalence of CAD. Secondly, luminal stenosis was visually assessed on ICA. Although visual estimation of CCTA and ICA images is most widely used in practical situation in China, using quantitative coronary angiography as standard reference may improve diagnostic performance of the DL algorithm. Thirdly, we only used the DL algorithm to diagnose anatomically significant CAD on CCTA. Since the issue of detecting functionally significant CAD (using fractional flow reserve derived from CCTA) has seen a recent explosion of interest (30–32), further studies will pay more attention to artificial intelligence in investigating hemodynamic alterations in CAD.

In conclusion, we used a completely external dataset to test the diagnostic performance of the DL algorithm. In this multi-center, multi-vendor study, we found that the DL algorithm worked faster than humans and performed no inferior to experts in terms of sensitivity, NPV and AUC. The AUCs of the DL algorithm remained satisfactory without significant group differences in patients stratified by sex, age and geographic area, as well as data stratified by CT scanner type. Our study indicated that the DL algorithm could benefit patients and clinicians due to its good accuracy, generalizability and time efficiency.

## Data Availability Statement

The original contributions presented in the study are included in the article/Supplementary Material, further inquiries can be directed to the corresponding author.

## Ethics Statement

The studies involving human participants were reviewed and approved by Capital Medical University Affiliated Beijing Friendship Hospital. Written informed consent was not obtained from the individual(s) for the publication of any potentially identifiable images or data included in this article.

## Author Contributions

All authors listed have made a substantial, direct and intellectual contribution to the work, and approved it for publication.

## Funding

This study received funding from National Key Research and Development Program of China (2019YFE0107800), Beijing Municipal Science and Technology Commission (Z201100005620009) to ZY, and National Research Foundation of Korea (2019K1A3A1A20093097) to MH. The funders had the following involvement with the study. All the funders provided financial support for patient enrollment, data collection, database construction, and management.

## Conflict of Interest

NG was employed by company Shukun (Beijing) Technology Co., Ltd. The remaining authors declare that the research was conducted in the absence of any commercial or financial relationships that could be construed as a potential conflict of interest.

## Publisher's Note

All claims expressed in this article are solely those of the authors and do not necessarily represent those of their affiliated organizations, or those of the publisher, the editors and the reviewers. Any product that may be evaluated in this article, or claim that may be made by its manufacturer, is not guaranteed or endorsed by the publisher.
