# Corrigendum: Diagnostic Accuracy and Generalizability of a Deep Learning-Based Fully Automated Algorithm for Coronary Artery Stenosis Detection on CCTA: A Multi-Centre Registry Study

**DOI:** 10.3389/fcvm.2022.920738

**Published:** 2022-06-13

**Authors:** Lixue Xu, Yi He, Nan Luo, Ning Guo, Min Hong, Xibin Jia, Zhenchang Wang, Zhenghan Yang

**Affiliations:** ^1^Affiliated Beijing Friendship Hospital, Capital Medical University, Beijing, China; ^2^Shukun (Beijing) Technology Co., Ltd., Beijing, China; ^3^Department of Computer Software Engineering, Soonchunhyang University, Asan-si, South Korea; ^4^Faculty of Information Technology, Beijing University of Technology, Beijing, China

**Keywords:** coronary artery disease, computed tomographic angiography, deep learning, invasive coronary angiography (ICA), diagnostic test

In the original article, we neglected to include the funder **“National Key Research and Development Program of China (2019YFE0107800), Beijing Municipal Science and Technology Commission (Z201100005620009) to ZY, and National Research Foundation of Korea (2019K1A3A1A20093097) to MH.”**

The correct funding statement appears below:

“This study received funding from National Key Research and Development Program of China (2019YFE0107800), Beijing Municipal Science and Technology Commission (Z201100005620009) to ZY, and National Research Foundation of Korea (2019K1A3A1A20093097) to MH. The funders had the following involvement with the study. All the funders provided financial support for patient enrollment, data collection, database construction, and management.”

The authors apologize for this error and state that this does not change the scientific conclusions of the article in any way. The original article has been updated.

## Publisher's Note

All claims expressed in this article are solely those of the authors and do not necessarily represent those of their affiliated organizations, or those of the publisher, the editors and the reviewers. Any product that may be evaluated in this article, or claim that may be made by its manufacturer, is not guaranteed or endorsed by the publisher.

