# Supplementary material for: Diagnostic Accuracy and Generalizability of a Deep Learning-Based Fully Automated Algorithm for Coronary Artery Stenosis Detection on CCTA: A Multi-Centre Registry Study
Source: Front Cardiovasc Med. 2021 Nov 5;8:707508. doi: 10.3389/fcvm.2021.707508 (PMC8602896; doi:10.3389/fcvm.2021.707508)
Supplement: Supplementary file 1 [file Data_Sheet_1.docx]

**Supplementary materials**

Supplementary Table S1. Hospitals in current registered study.

| Site no. | City and province | Areas | *n* |
| --- | --- | --- | --- |
| 1 | Hangzhou City, Zhejiang Province | East | 24 |
| 2 | Shaoxing City, Zhejiang Province | East | 20 |
| 3 | Jinhua City, Zhejiang Province | East | 28 |
| 4 | Nanjing City, Jiangsu Province | East | 31 |
| 5 | Nanjing City Jiangsu Province | East | 14 |
| 6 | Nanchang City, Jiangxi Province | East | 3 |
| 7 | Jilin City, Jilin Province | Northeast | 25 |
| 8 | Harbin City, Heilongjiang Province | Northeast | 22 |
| 9 | Dalian City, Liaoning Province | Northeast | 16 |
| 10 | Lanzhou City, Gansu Province | Northwest | 28 |
| 11 | Lanzhou City, Gansu Province | Northwest | 22 |
| 12 | Yinchuan City, Ningxia Province | Northwest | 20 |
| 13 | Xi’an City, Shaanxi Province | Northwest | 13 |
| 14 | Xi’an City, Shaanxi Province | Northwest | 17 |
| 15 | Beijing City | North | 48 |
| 16 | Beijing City | North | 13 |
| 17 | Jincheng City, Shanxi Province | North | 35 |
| 18 | Baoding City, Hebei Province | North | 11 |
| 19 | Huhhot City, Inner Mongolia Autonomous Region | North | 18 |
| 20 | Huhhot City, Inner Mongolia Autonomous Region | North | 21 |
| 21 | Tianjin City | North | 20 |
| 22 | Changsha City, Hunan Province | South | 6 |
| 23 | Nanning City, Guangxi Province | South | 7 |
| 24 | Shenzhen City | South | 24 |
| 25 | Zhangzhou City, Fujian Province | South | 20 |
| 26 | Xiamen City, Fujian Province | South | 5 |
| 27 | Kunming City, Yunnan Province | South | 16 |

Since the imbalanced sample size may interfere the results, we classified the sites from Middle China (site no.22), South China(site no.23,24,25,26) and Southwest of China (site no.27) as ‘South’, according to their close geographical location.

Supplementary Table S2. Baseline information of 5 geographic areas.

|  | Northeast | North | East | Northwest | South | *P* value |
| --- | --- | --- | --- | --- | --- | --- |
|  | *n*=63 | *n*=166 | *n*=120 | *n*=100 | *n*=78 |  |
| Sex |  |  |  |  |  | 0.061 |
| Male | 34/63(54.0%) | 108/166(65.1%) | 81/120(67.5%) | 70/100(70.0%) | 60/78(76.9%) |  |
| Female | 29/63(46.0%) | 58/166(34.9%) | 39/120(32.5%) | 30/100(30.0%) | 18/78(23.1%) |  |
| Age (years) | 61.9±9.1 | 60.9±9.7 | 64.5±10.0 | 62.2±10.0 | 61.4±12.1 | 0.047* |
| HR (bpm) | 73.0±12.7 | 64.6±17.5 | 67.1±8.2 | 62.9±13.7 | 67.3±13.7 | 0.001* |
| BMI (kg/m^2^) | 23.6±2.5 | 25.8±3.0 | 24.0±2.9 | 24.6±2.7 | 23.0±3.0 | <0.001* |
| Hypertension | 36/59(61.0%) | 105/160(65.6%) | 69/116(59.5%) | 51/96(53.1%) | 49/76(64.5%) | 0.351 |
| Hyperlipidemia | 16/53(30.2%) | 34/157(21.7%) | 6/93(6.5%) | 7/90(7.8%) | 18/71(25.4%) | <0.001* |
| DM | 17/59(28.8%) | 53/160(33.1%) | 26/117(22.2%) | 18/91(19.8%) | 19/70(27.1%) | 0.140 |
| Smoking | 19/46(41.3%) | 69/152(45.4%) | 42/116(36.2%) | 44/100(44.0%) | 33/76(43.4%) | 0.578 |
| CAD history | 3/60(5.0%) | 14/162(8.6%) | 4/117(3.4%) | 1/98(1.0%) | 2/76(2.6%) | 0.044* |
| Tube potential |  |  |  |  |  | <0.001* |
| ≤80kV | 2/63(3.2%) | - | 12/120(10.0%) | - | 3/78(3.8%) |  |
| 90~110kV | 20/63(31.7%) | 35/166(21.1%) | 45/120(37.5) | 25/100(25.0%) | 53/78(67.9%) |  |
| ≥120kV | 41/66(65.1%) | 131/166(78.9%) | 63/120(52.5%) | 75/100(75.0%) | 22/78(28.2%) |  |
| DLP (mSv×cm) | 889.4±239.6 | 541.5±315.4 | 698.4±463.8 | 559.9±371.7 | 657.5±457.5 |  |

Data are n/N (% of non-missing data) or mean (SD).

Abbreviation: HR=hear rate; BMI=body mass index; DM=diabetes mellitus; CAD=coronary artery disease. DLP=dose length product.

*indicates a significant p<0.05.


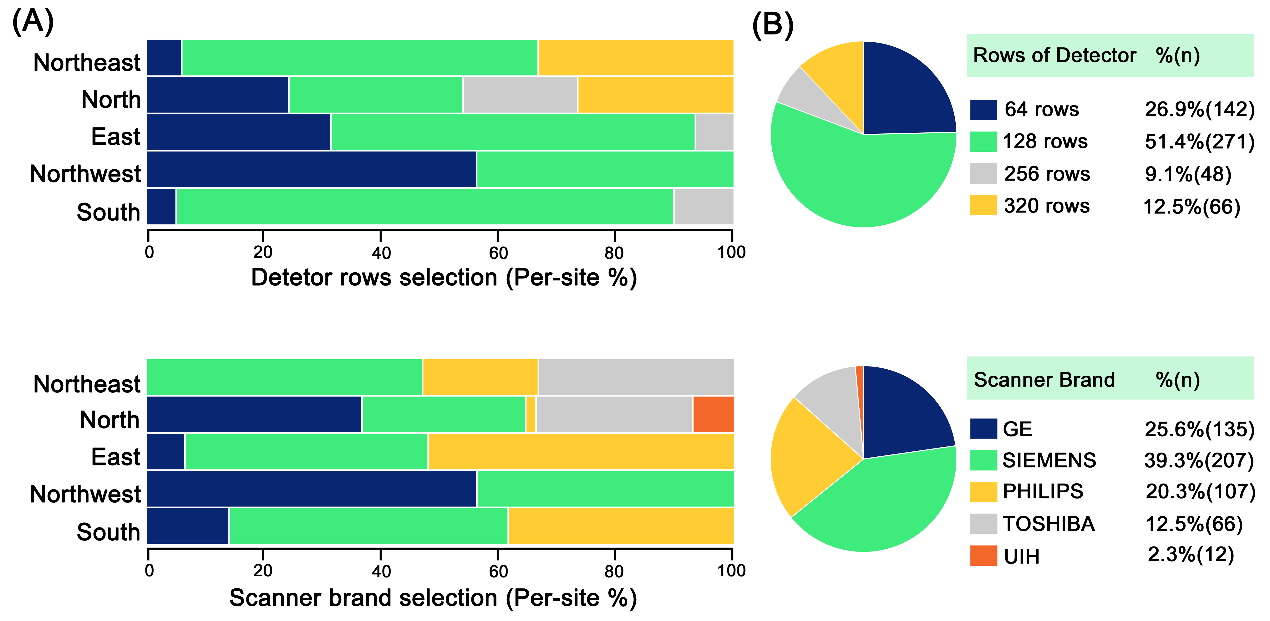


Supplementary Figure S1. Application of types of CT scanners in current study.


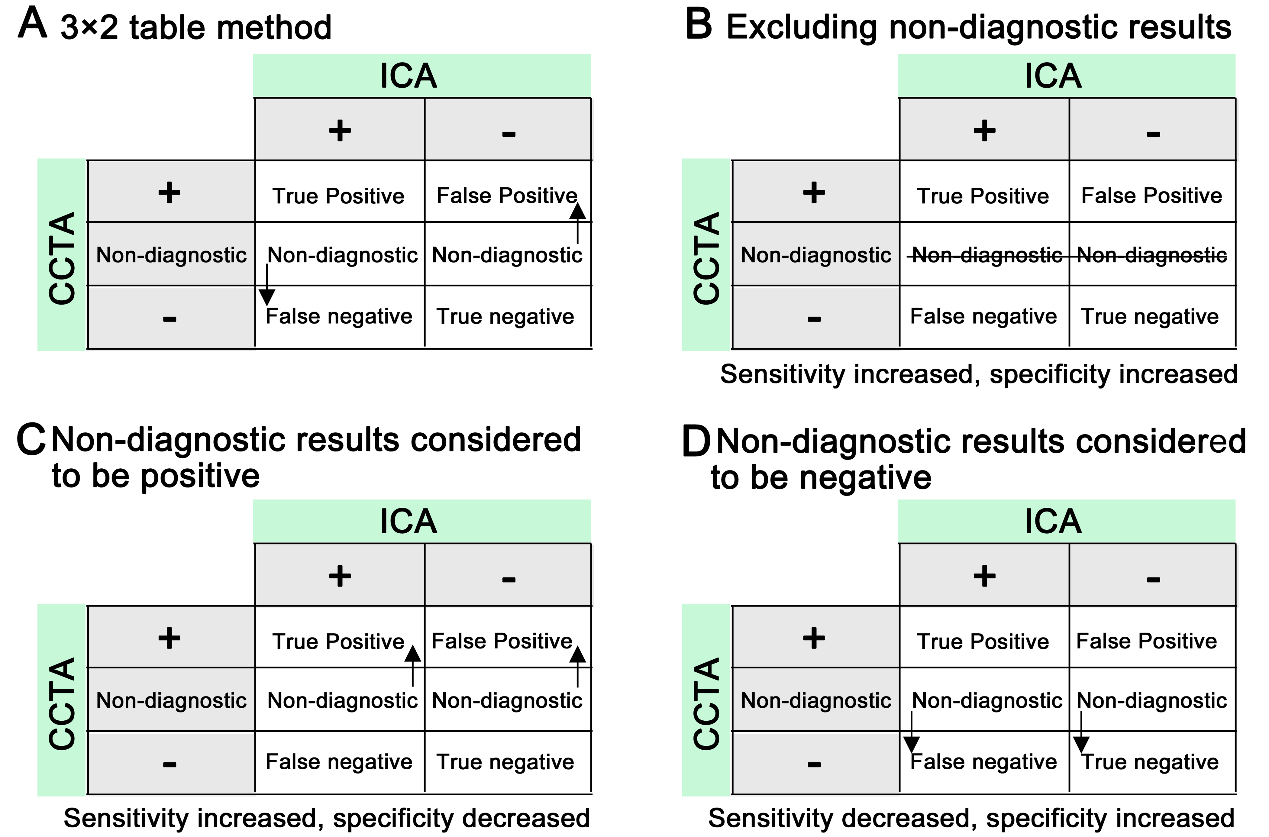


Supplementary Figure S2. Different methods of handling non-diagnostic results.


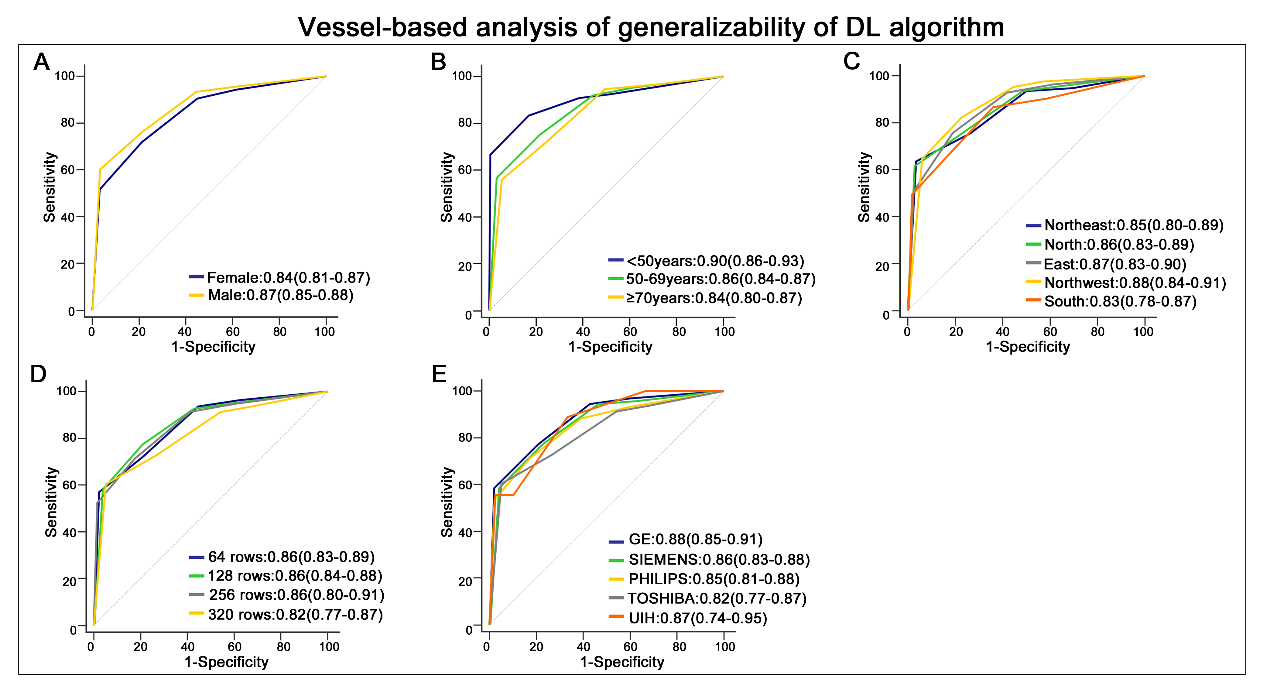


Supplementary Figure S3**.** Robustness of the deep learning (DL)-based fully automated algorithm at vessel-based level (≥50% diameter stenosis was defined as CAD).

(A-E) displays the receiver operating characteristic (ROC) curve of the DL algorithm in different subgroups at the vessel-based level. (A) shows the results of males and females, (B) is the outcome of patients with different ages, (C) depicts the results of patients from different geographic areas, and (D) and (E) display the outcomes of data acquired on different rows of detectors and different brands of CT scanners.


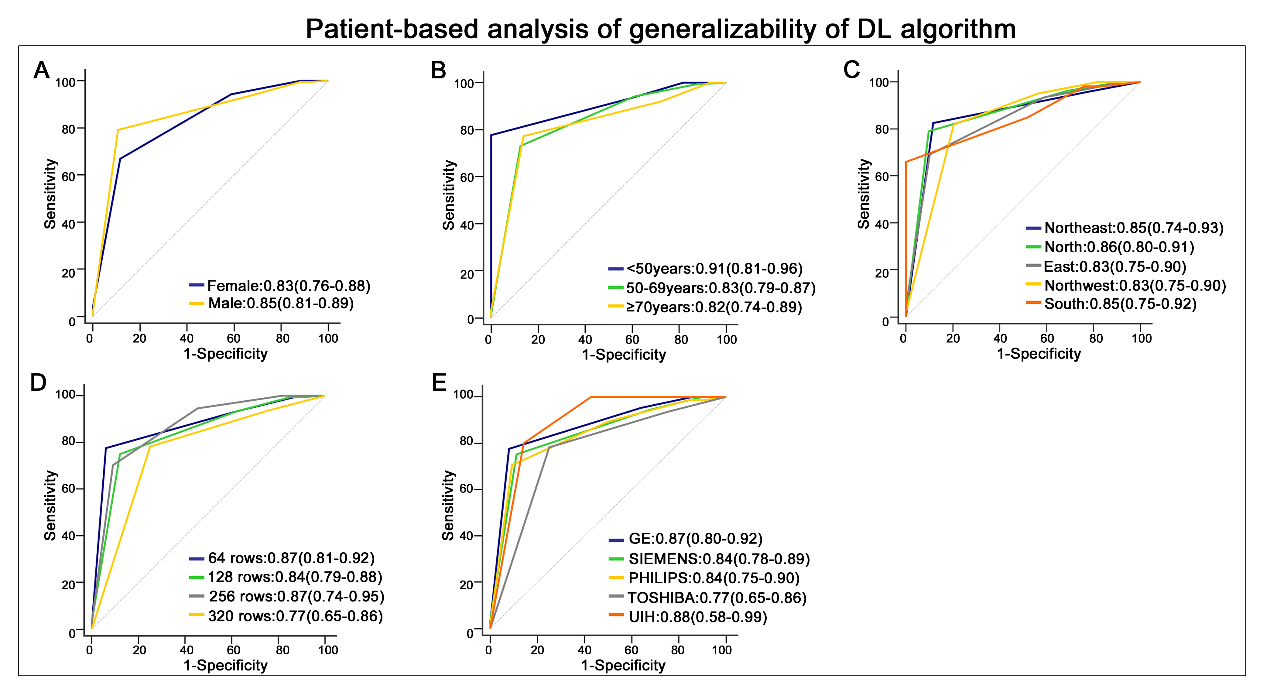


Supplementary Figure S4**.** Robustness of the deep learning (DL)-based fully automated algorithm at patient-based level (≥50% diameter stenosis was defined as CAD).

(A-E) displays the receiver operating characteristic (ROC) curve of the DL algorithm in different subgroups at the patient-based level. (A) shows the results of males and females, (B) is the outcome of patients with different ages, (C) depicts the results of patients from different geographic areas, and (D) and (E) display the outcomes of data acquired on different rows of detectors and different brands of CT scanners.
